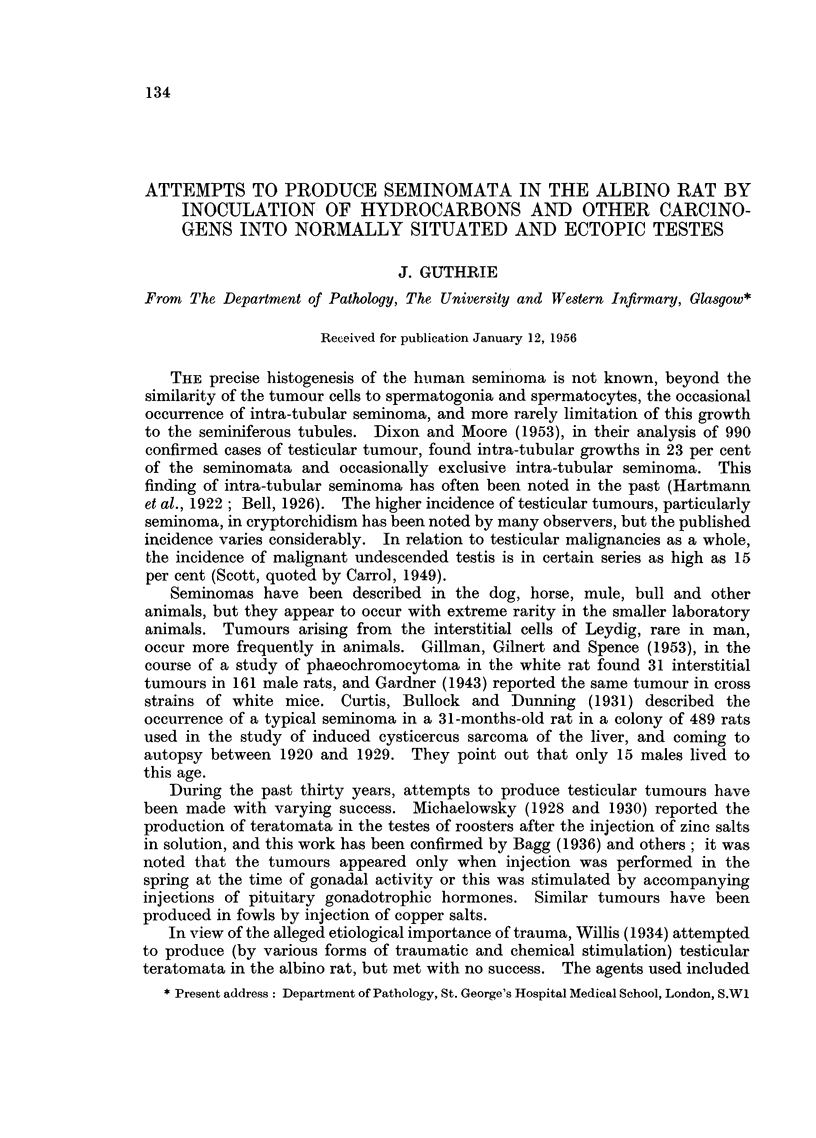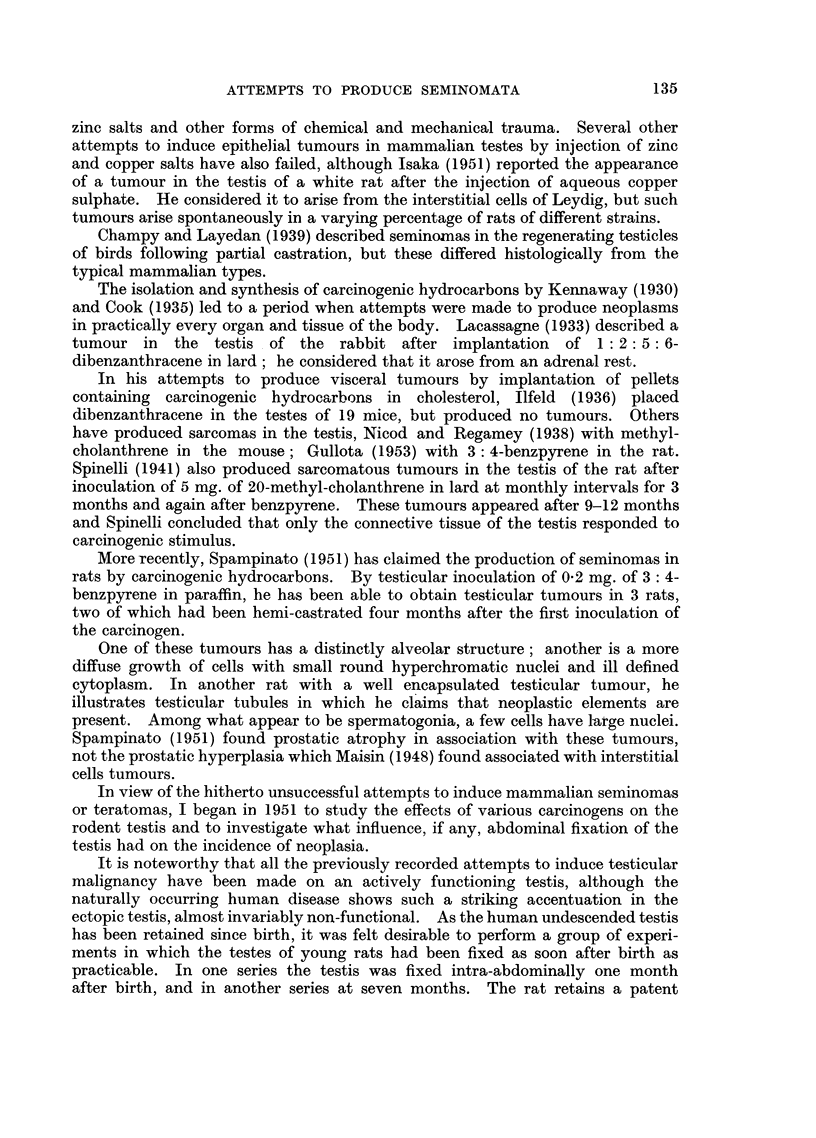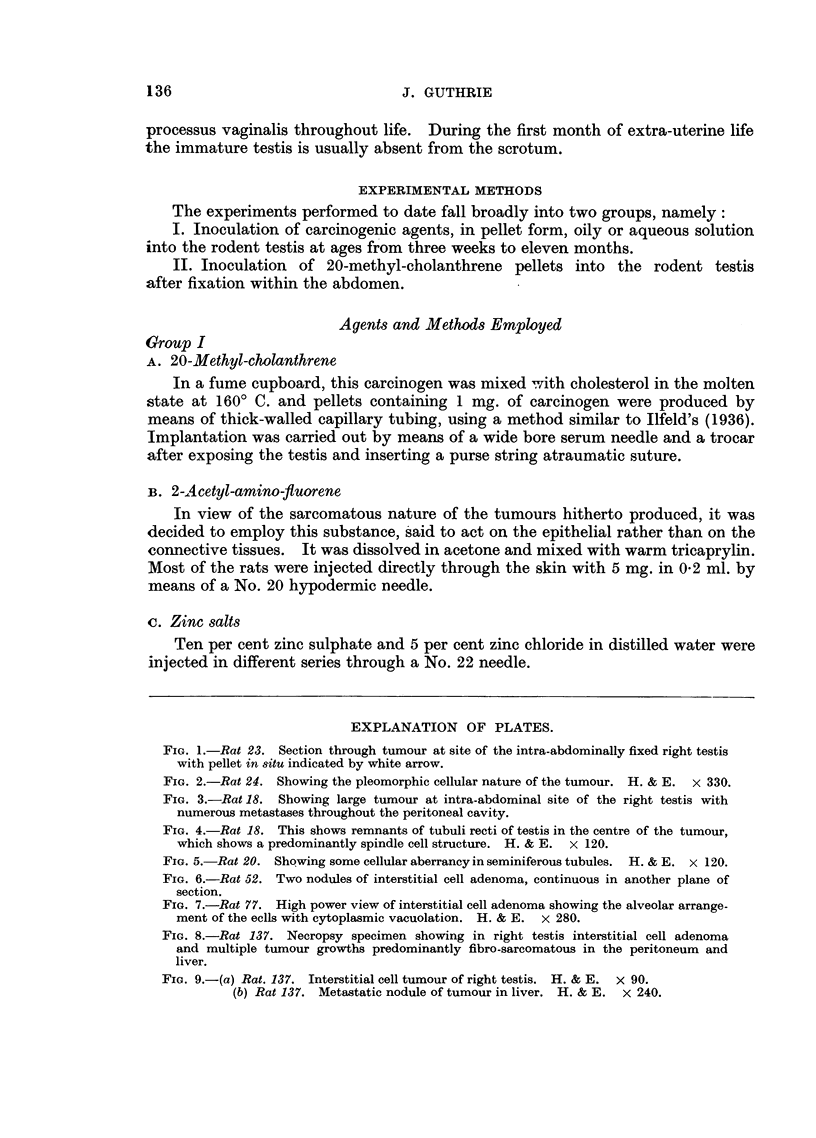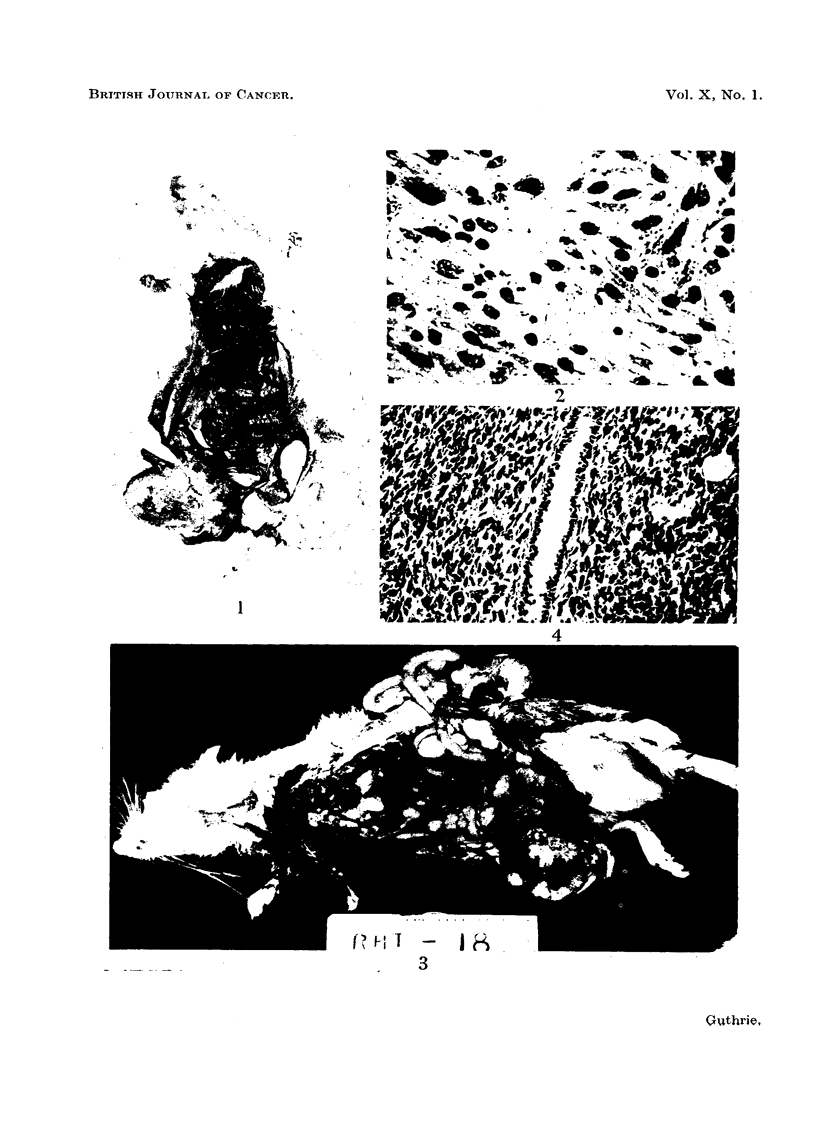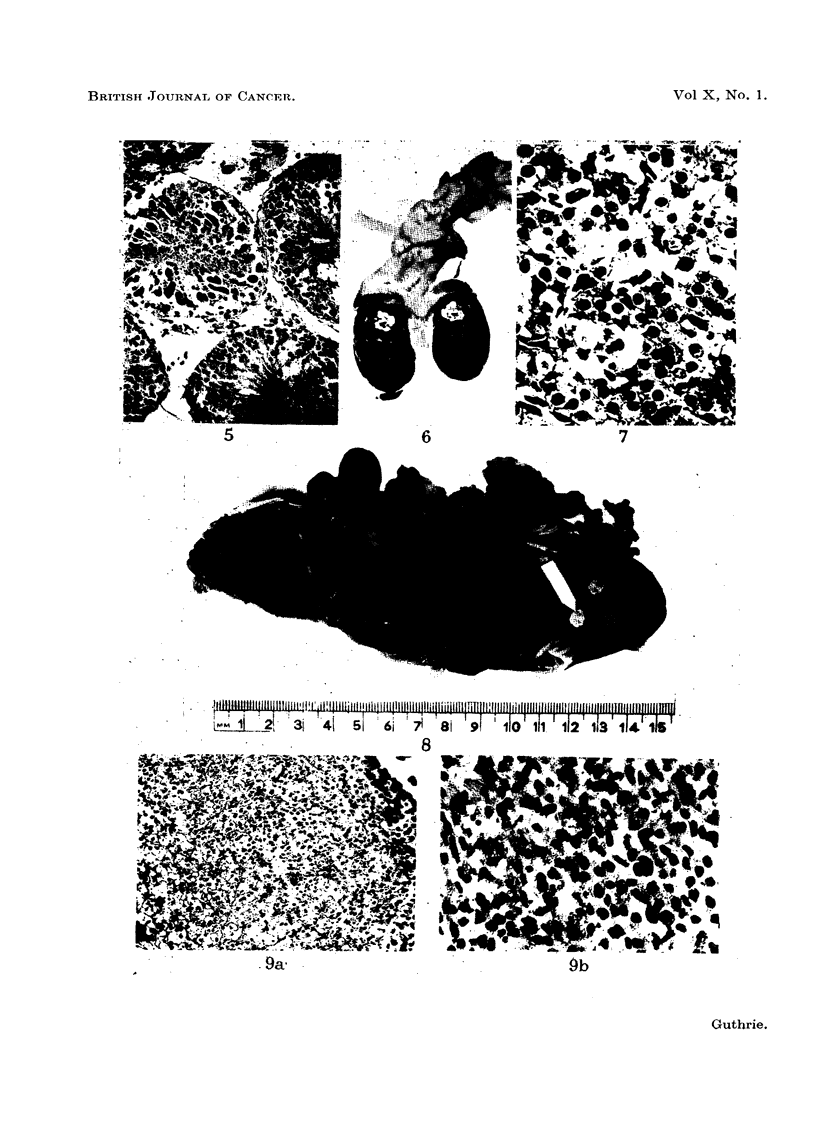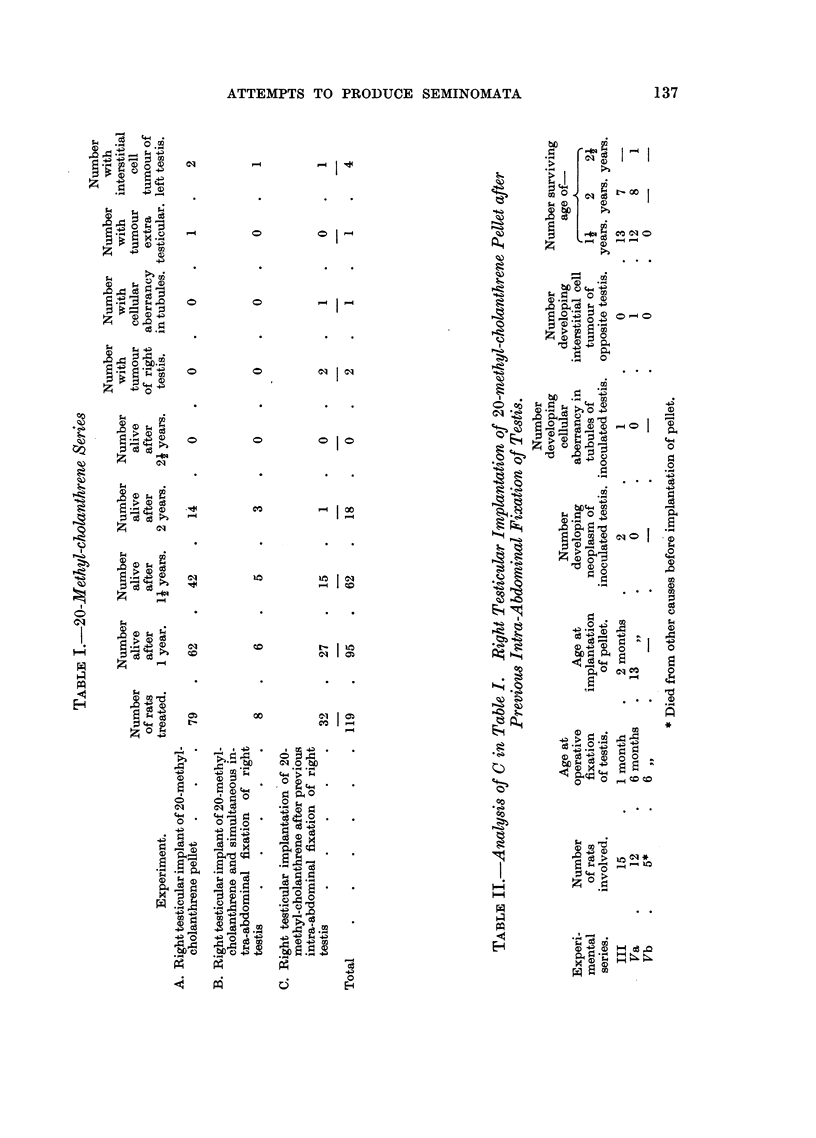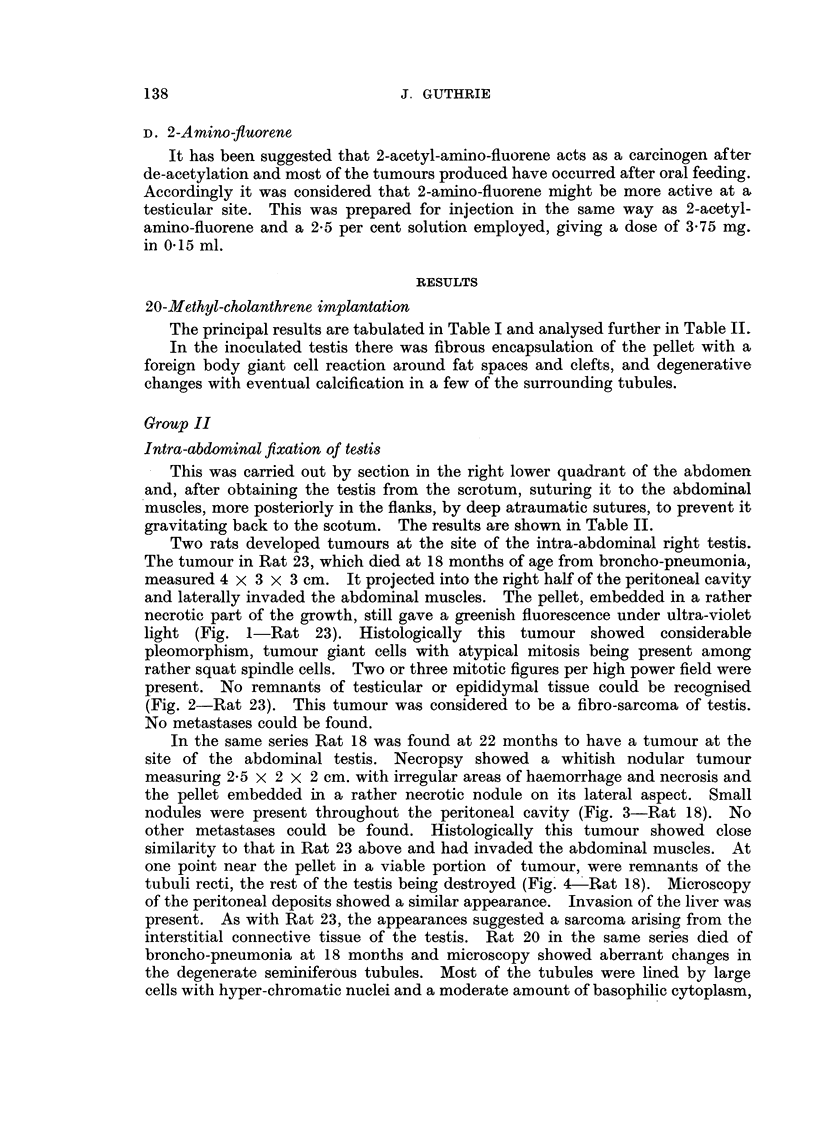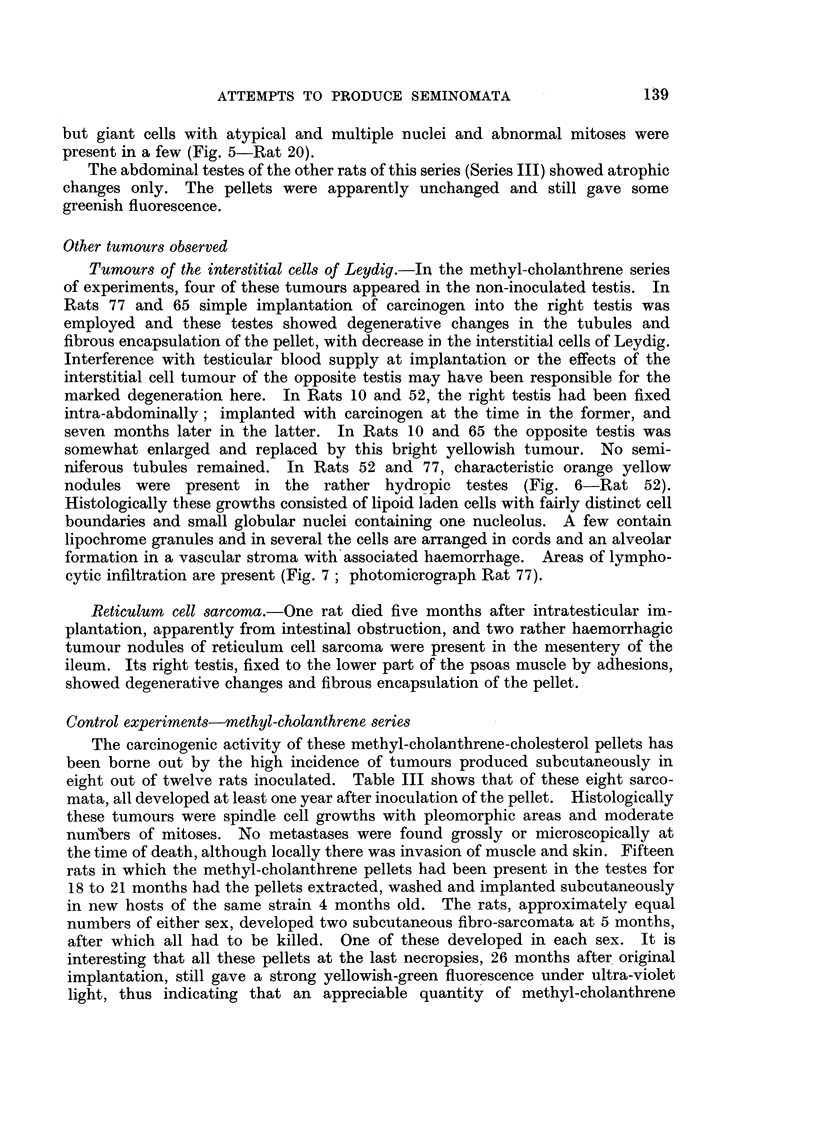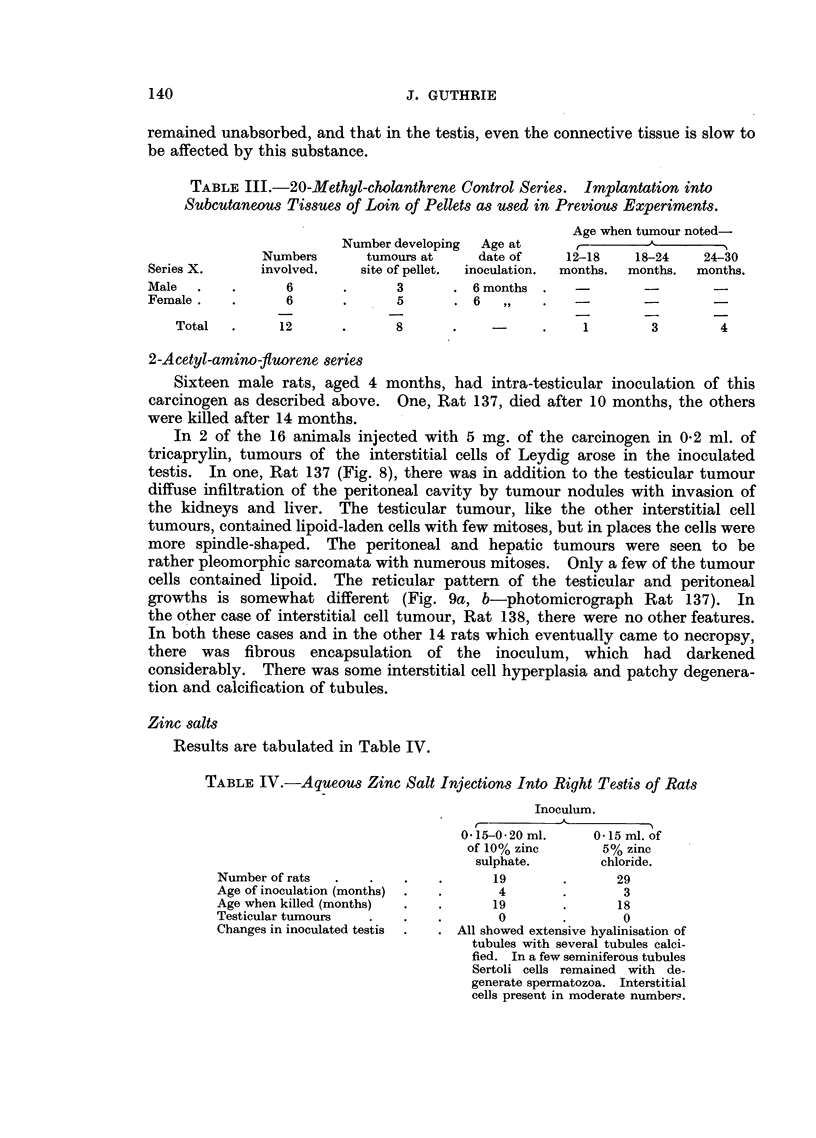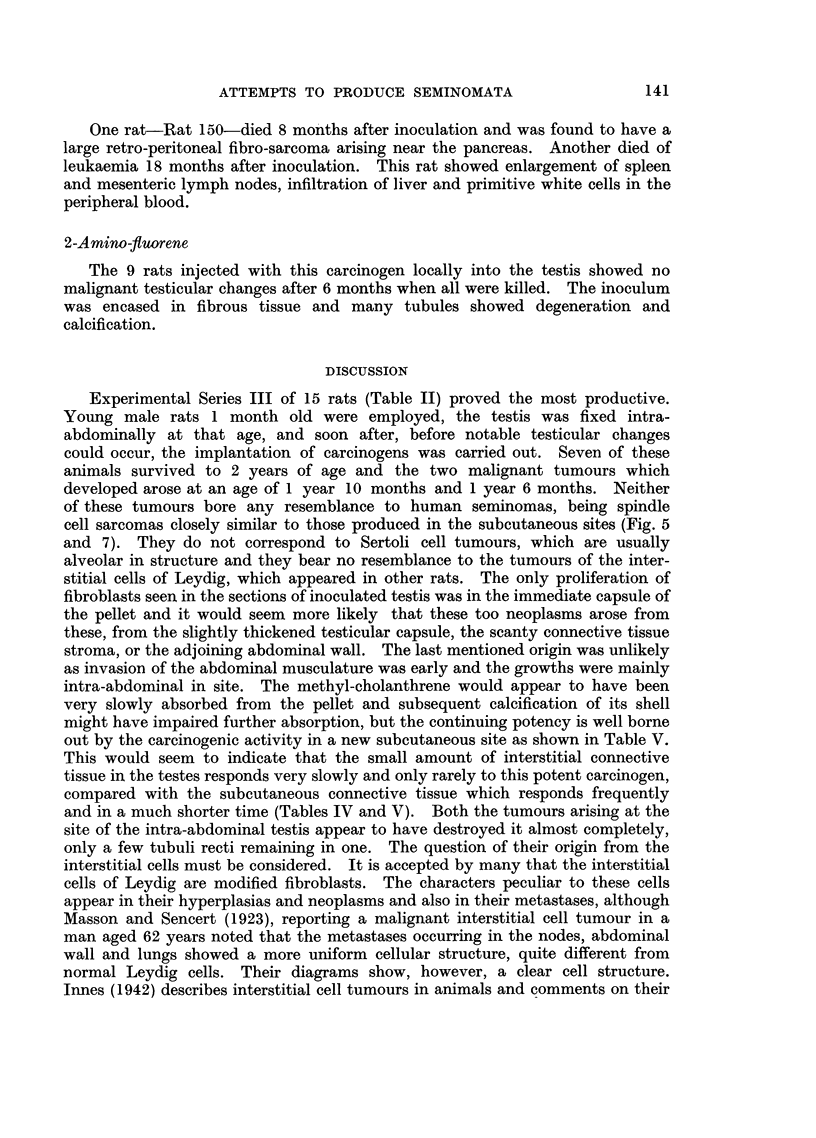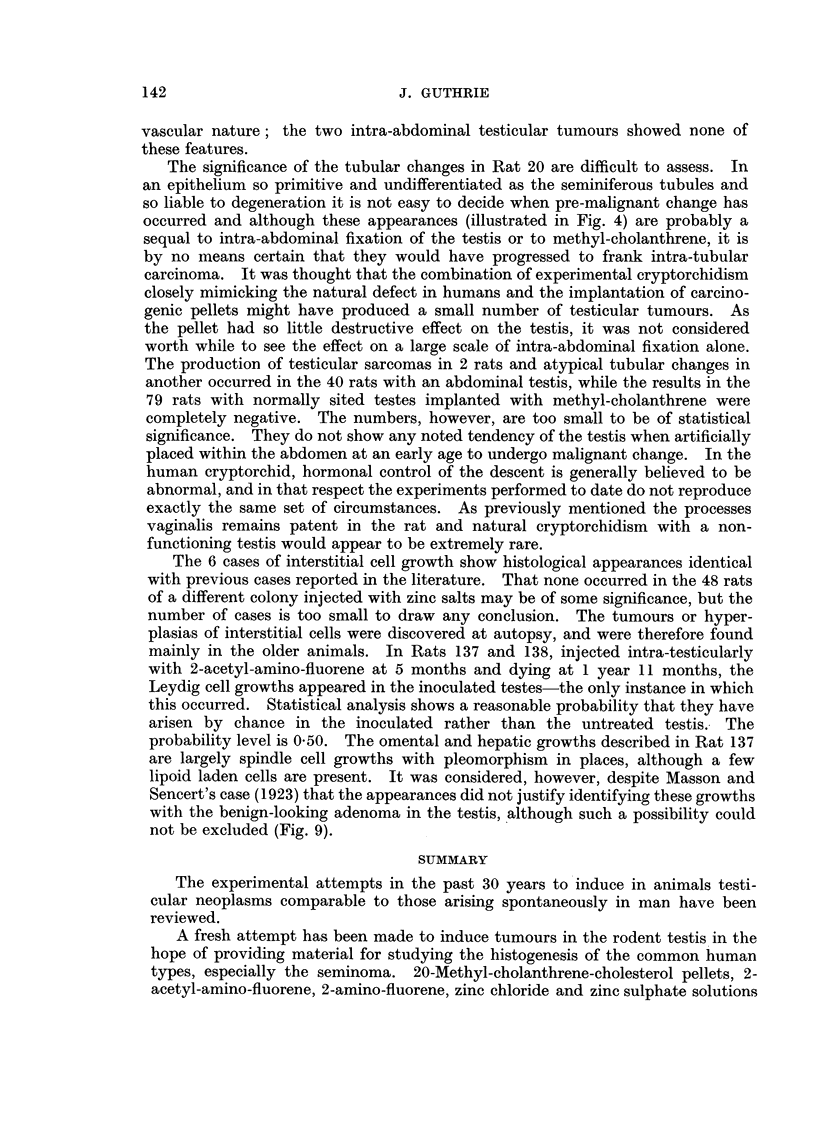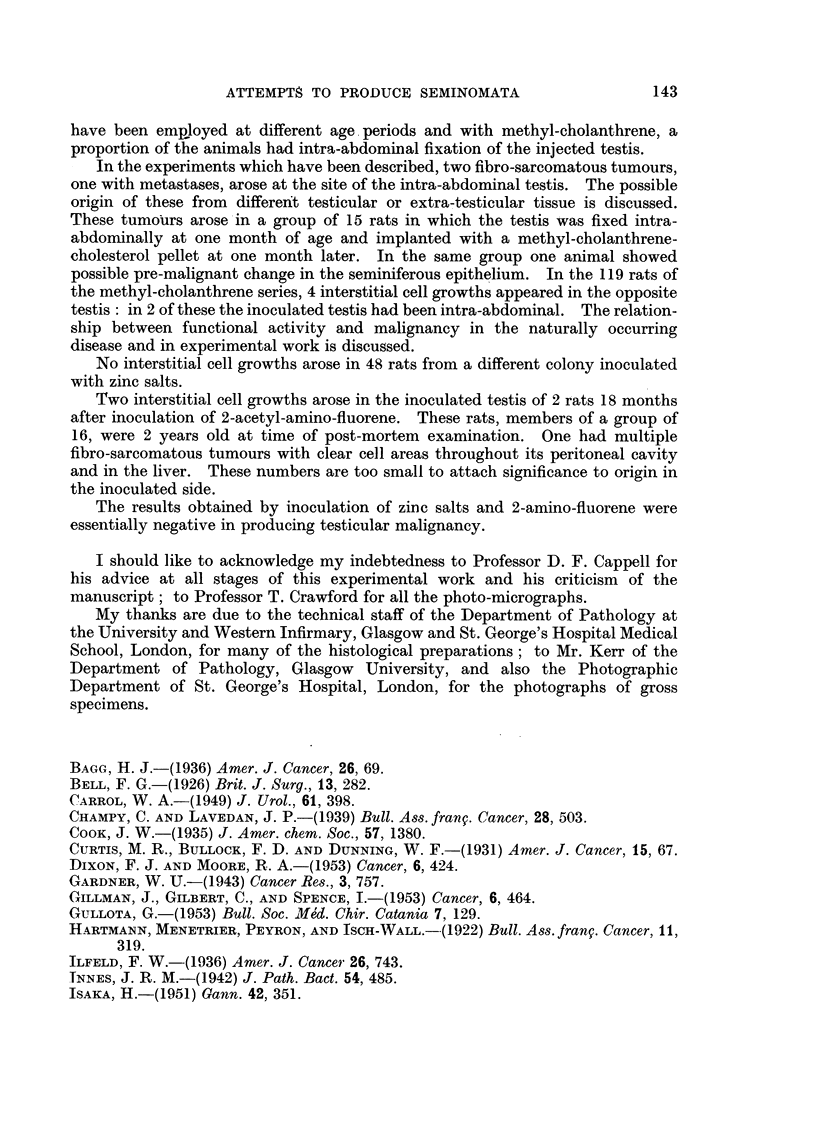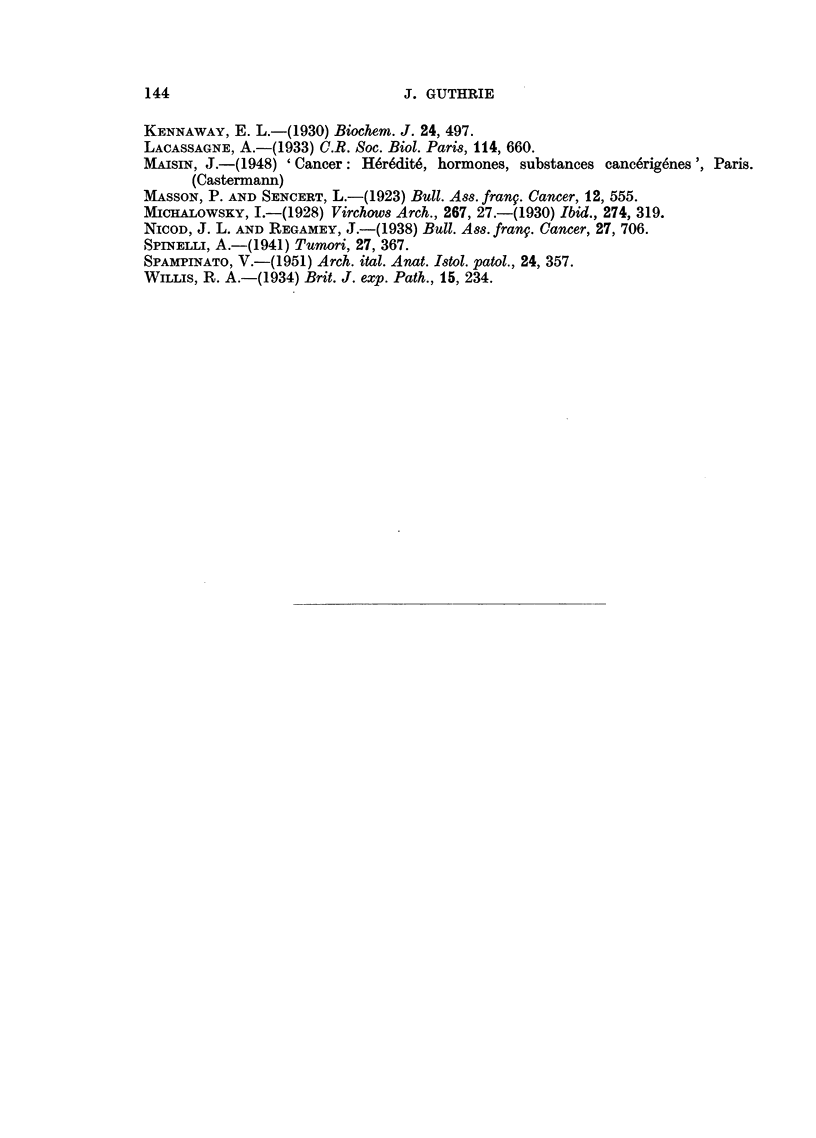# Attempts to Produce Seminomata in the Albino Rat by Inoculation of Hydrocarbons and other Carcinogens into Normally Situated and Ectopic Testes

**DOI:** 10.1038/bjc.1956.18

**Published:** 1956-03

**Authors:** J. Guthrie

## Abstract

**Images:**


### ATTEMPTS TO PRODUCE SEMINOMATA IN THE ALBINO RAT BY INOCULATION OF HYDROCARBONS AND OTHER CARCINOGENS INTO NORMALLY SITUATED AND ECTOPIC TESTES

J. GUTHRIE

*From The Department of Pathology, The University and Western Infirmary, Glasgow**

Received for publication January 12, 1956

THE precise histogenesis of the human seminoma is not known, beyond the similarity of the tumour cells to spermatogonia and spermatocytes, the occasional occurrence of intra-tubular seminoma, and more rarely limitation of this growth to the seminiferous tubules. Dixon and Moore (1953), in their analysis of 990 confirmed cases of testicular tumour, found intra-tubular growths in 23 per cent of the seminomata and occasionally exclusive intra-tubular seminoma. This finding of intra-tubular seminoma has often been noted in the past (Hartmann *et al.*, 1922 ; Bell, 1926). The higher incidence of testicular tumours, particularly seminoma, in cryptorchidism has been noted by many observers, but the published incidence varies considerably. In relation to testicular malignancies as a whole, the incidence of malignant undescended testis is in certain series as high as 15 per cent (Scott, quoted by Carrol, 1949).

Seminomas have been described in the dog, horse, mule, bull and other animals, but they appear to occur with extreme rarity in the smaller laboratory animals. Tumours arising from the interstitial cells of Leydig, rare in man, occur more frequently in animals. Gillman, Gilnert and Spence (1953), in the course of a study of phaeochromocytoma in the white rat found 31 interstitial tumours in 161 male rats, and Gardner (1943) reported the same tumour in cross strains of white mice. Curtis, Bullock and Dunning (1931) described the occurrence of a typical seminoma in a 31-months-old rat in a colony of 489 rats used in the study of induced cysticercus sarcoma of the liver, and coming to autopsy between 1920 and 1929. They point out that only 15 males lived to this age.

During the past thirty years, attempts to produce testicular tumours have been made with varying success. Michaelowsky (1928 and 1930) reported the production of teratomata in the testes of roosters after the injection of zinc salts in solution, and this work has been confirmed by Bagg (1936) and others ; it was noted that the tumours appeared only when injection was performed in the spring at the time of gonadal activity or this was stimulated by accompanying injections of pituitary gonadotrophic hormones. Similar tumours have been produced in fowls by injection of copper salts.

In view of the alleged etiological importance of trauma, Willis (1934) attempted to produce (by various forms of traumatic and chemical stimulation) testicular teratomata in the albino rat, but met with no success. The agents used included

* Present address : Department of Pathology, St. George's Hospital Medical School, London, S.W1

zinc salts and other forms of chemical and mechanical trauma. Several other attempts to induce epithelial tumours in mammalian testes by injection of zinc and copper salts have also failed, although Isaka (1951) reported the appearance of a tumour in the testis of a white rat after the injection of aqueous copper sulphate. He considered it to arise from the interstitial cells of Leydig, but such tumours arise spontaneously in a varying percentage of rats of different strains.

Champy and Layedan (1939) described seminomas in the regenerating testicles of birds following partial castration, but these differed histologically from the typical mammalian types.

The isolation and synthesis of carcinogenic hydrocarbons by Kennaway (1930) and Cook (1935) led to a period when attempts were made to produce neoplasms in practically every organ and tissue of the body. Lacassagne (1933) described a tumour in the testis of the rabbit after implantation of 1 : 2 : 5 : 6-dibenzanthracene in lard ; he considered that it arose from an adrenal rest.

In his attempts to produce visceral tumours by implantation of pellets containing carcinogenic hydrocarbons in cholesterol, Ilfeld (1936) placed dibenzanthracene in the testes of 19 mice, but produced no tumours. Others have produced sarcomas in the testis, Nicod and Regamey (1938) with methyl-cholanthrene in the mouse ; Gullota (1953) with 3 : 4-benzpyrene in the rat. Spinelli (1941) also produced sarcomatous tumours in the testis of the rat after inoculation of 5 mg. of 20-methyl-cholanthrene in lard at monthly intervals for 3 months and again after benzpyrene. These tumours appeared after 9–12 months and Spinelli concluded that only the connective tissue of the testis responded to carcinogenic stimulus.

More recently, Spampinato (1951) has claimed the production of seminomas in rats by carcinogenic hydrocarbons. By testicular inoculation of 0·2 mg. of 3 : 4-benzpyrene in paraffin, he has been able to obtain testicular tumours in 3 rats, two of which had been hemi-castrated four months after the first inoculation of the carcinogen.

One of these tumours has a distinctly alveolar structure ; another is a more diffuse growth of cells with small round hyperchromatic nuclei and ill defined cytoplasm. In another rat with a well encapsulated testicular tumour, he illustrates testicular tubules in which he claims that neoplastic elements are present. Among what appear to be spermatogonia, a few cells have large nuclei. Spampinato (1951) found prostatic atrophy in association with these tumours, not the prostatic hyperplasia which Maisin (1948) found associated with interstitial cells tumours.

In view of the hitherto unsuccessful attempts to induce mammalian seminomas or teratomas, I began in 1951 to study the effects of various carcinogens on the rodent testis and to investigate what influence, if any, abdominal fixation of the testis had on the incidence of neoplasia.

It is noteworthy that all the previously recorded attempts to induce testicular malignancy have been made on an actively functioning testis, although the naturally occurring human disease shows such a striking accentuation in the ectopic testis, almost invariably non-functional. As the human undescended testis has been retained since birth, it was felt desirable to perform a group of experiments in which the testes of young rats had been fixed as soon after birth as practicable. In one series the testis was fixed intra-abdominally one month after birth, and in another series at seven months. The rat retains a patent processus vaginalis throughout life. During the first month of extra-uterine life the immature testis is usually absent from the scrotum.

### EXPERIMENTAL METHODS

The experiments performed to date fall broadly into two groups, namely:

I. Inoculation of carcinogenic agents, in pellet form, oily or aqueous solution into the rodent testis at ages from three weeks to eleven months.

II. Inoculation of 20-methyl-cholanthrene pellets into the rodent testis after fixation within the abdomen.

### *Agents and Methods Employed*

*Group I*

A. 20-*Methyl-cholanthrene*

In a fume cupboard, this carcinogen was mixed with cholesterol in the molten state at 160° C. and pellets containing 1 mg. of carcinogen were produced by means of thick-walled capillary tubing, using a method similar to Ilfeld's (1936). Implantation was carried out by means of a wide bore serum needle and a trocar after exposing the testis and inserting a purse string atraumatic suture.

B. 2-*Acetyl-amino-fluorene*

In view of the sarcomatous nature of the tumours hitherto produced, it was decided to employ this substance, said to act on the epithelial rather than on the connective tissues. It was dissolved in acetone and mixed with warm tricaprylin. Most of the rats were injected directly through the skin with 5 mg. in 0·2 ml. by means of a No. 20 hypodermic needle.

C. *Zinc salts*

Ten per cent zinc sulphate and 5 per cent zinc chloride in distilled water were injected in different series through a No. 22 needle.

---

EXPLANATION OF PLATES.

FIG. 1.—*Rat 23.* Section through tumour at site of the intra-abdominally fixed right testis with pellet *in situ* indicated by white arrow.

FIG. 2.—*Rat 24.* Showing the pleomorphic cellular nature of the tumour. H. & E. × 330.

FIG. 3.—*Rat 18.* Showing large tumour at intra-abdominal site of the right testis with numerous metastases throughout the peritoneal cavity.

FIG. 4.—*Rat 18.* This shows remnants of tubuli recti of testis in the centre of the tumour, which shows a predominantly spindle cell structure. H. & E. × 120.

FIG. 5.—*Rat 20.* Showing some cellular aberrancy in seminiferous tubules. H. & E. × 120.

FIG. 6.—*Rat 52.* Two nodules of interstitial cell adenoma, continuous in another plane of section.

FIG. 7.—*Rat 77.* High power view of interstitial cell adenoma showing the alveolar arrangement of the eclls with cytoplasmic vacuolation. H. & E. × 280.

FIG. 8.—*Rat 137.* Necropsy specimen showing in right testis interstitial cell adenoma and multiple tumour growths predominantly fibro-sarcomatous in the peritoneum and liver.

FIG. 9.—(*a*) *Rat. 137.* Interstitial cell tumour of right testis. H. & E. × 90.
(*b*) *Rat 137.* Metastatic nodule of tumour in liver. H. & E. × 240.

1

2

4

3

5

6

7

8

9a

9b

Guthrie.

TABLE I.—*20-Methyl-cholanthrene Series*

| Experiment. | Number of rats treated. | Number alive after 1 year. | Number alive after 1½ years. | Number alive after 2 years. | Number alive after 2½ years. | Number with tumour of right testis. | Number with cellular aberrancy in tubules. | Number with tumour extra testicular. | Number with interstitial cell tumour of left testis. |
|---|---|---|---|---|---|---|---|---|---|
| A. Right testicular implant of 20-methyl-cholanthrene pellet | 79 | 62 | 42 | 14 | 0 | 0 | 0 | 1 | 2 |
| B. Right testicular implant of 20-methyl-cholanthrene and simultaneous intra-abdominal fixation of right testis | 8 | 6 | 5 | 3 | 0 | 0 | 0 | 0 | 1 |
| C. Right testicular implantation of 20-methyl-cholanthrene after previous intra-abdominal fixation of right testis | 32 | 27 | 15 | 1 | 0 | 2 | 1 | 0 | 1 |
| Total | 119 | 95 | 62 | 18 | 0 | 2 | 1 | 1 | 4 |

TABLE II.—*Analysis of C in Table I. Right Testicular Implantation of 20-methyl-cholanthrene Pellet after Previous Intra-Abdominal Fixation of Testis.*

| Experimental series. | Number of rats involved. | Age at operative fixation of testis. | Age at implantation of pellet. | Number developing neoplasm of inoculated testis. | Number developing cellular aberrancy in tubules of inoculated testis. | Number developing interstitial cell tumour of opposite testis. | Number surviving age of— 1½ years. | 2 years. | 2½ years. |
|---|---|---|---|---|---|---|---|---|---|
| III | 15 | 1 month | 2 months | 2 | 1 | 0 | 13 | 7 | — |
| *V*a | 12 | 6 months | 13 ,, | 0 | 0 | 1 | 12 | 8 | 1 |
| *V*b | 5* | 6 ,, | — | — | — | 0 | 0 | — | — |

* Died from other causes before implantation of pellet.

D. 2-*Amino-fluorene*

It has been suggested that 2-acetyl-amino-fluorene acts as a carcinogen after de-acetylation and most of the tumours produced have occurred after oral feeding. Accordingly it was considered that 2-amino-fluorene might be more active at a testicular site. This was prepared for injection in the same way as 2-acetyl-amino-fluorene and a 2·5 per cent solution employed, giving a dose of 3·75 mg. in 0·15 ml.

### RESULTS

20-*Methyl-cholanthrene implantation*

The principal results are tabulated in Table I and analysed further in Table II.

In the inoculated testis there was fibrous encapsulation of the pellet with a foreign body giant cell reaction around fat spaces and clefts, and degenerative changes with eventual calcification in a few of the surrounding tubules.

*Group II*

*Intra-abdominal fixation of testis*

This was carried out by section in the right lower quadrant of the abdomen and, after obtaining the testis from the scrotum, suturing it to the abdominal muscles, more posteriorly in the flanks, by deep atraumatic sutures, to prevent it gravitating back to the scotum. The results are shown in Table II.

Two rats developed tumours at the site of the intra-abdominal right testis. The tumour in Rat 23, which died at 18 months of age from broncho-pneumonia, measured 4 × 3 × 3 cm. It projected into the right half of the peritoneal cavity and laterally invaded the abdominal muscles. The pellet, embedded in a rather necrotic part of the growth, still gave a greenish fluorescence under ultra-violet light (Fig. 1—Rat 23). Histologically this tumour showed considerable pleomorphism, tumour giant cells with atypical mitosis being present among rather squat spindle cells. Two or three mitotic figures per high power field were present. No remnants of testicular or epididymal tissue could be recognised (Fig. 2—Rat 23). This tumour was considered to be a fibro-sarcoma of testis. No metastases could be found.

In the same series Rat 18 was found at 22 months to have a tumour at the site of the abdominal testis. Necropsy showed a whitish nodular tumour measuring 2·5 × 2 × 2 cm. with irregular areas of haemorrhage and necrosis and the pellet embedded in a rather necrotic nodule on its lateral aspect. Small nodules were present throughout the peritoneal cavity (Fig. 3—Rat 18). No other metastases could be found. Histologically this tumour showed close similarity to that in Rat 23 above and had invaded the abdominal muscles. At one point near the pellet in a viable portion of tumour, were remnants of the tubuli recti, the rest of the testis being destroyed (Fig. 4—Rat 18). Microscopy of the peritoneal deposits showed a similar appearance. Invasion of the liver was present. As with Rat 23, the appearances suggested a sarcoma arising from the interstitial connective tissue of the testis. Rat 20 in the same series died of broncho-pneumonia at 18 months and microscopy showed aberrant changes in the degenerate seminiferous tubules. Most of the tubules were lined by large cells with hyper-chromatic nuclei and a moderate amount of basophilic cytoplasm, but giant cells with atypical and multiple nuclei and abnormal mitoses were present in a few (Fig. 5—Rat 20).

The abdominal testes of the other rats of this series (Series III) showed atrophic changes only. The pellets were apparently unchanged and still gave some greenish fluorescence.

*Other tumours observed*

*Tumours of the interstitial cells of Leydig.*—In the methyl-cholanthrene series of experiments, four of these tumours appeared in the non-inoculated testis. In Rats 77 and 65 simple implantation of carcinogen into the right testis was employed and these testes showed degenerative changes in the tubules and fibrous encapsulation of the pellet, with decrease in the interstitial cells of Leydig. Interference with testicular blood supply at implantation or the effects of the interstitial cell tumour of the opposite testis may have been responsible for the marked degeneration here. In Rats 10 and 52, the right testis had been fixed intra-abdominally ; implanted with carcinogen at the time in the former, and seven months later in the latter. In Rats 10 and 65 the opposite testis was somewhat enlarged and replaced by this bright yellowish tumour. No seminiferous tubules remained. In Rats 52 and 77, characteristic orange yellow nodules were present in the rather hydropic testes (Fig. 6—Rat 52). Histologically these growths consisted of lipoid laden cells with fairly distinct cell boundaries and small globular nuclei containing one nucleolus. A few contain lipochrome granules and in several the cells are arranged in cords and an alveolar formation in a vascular stroma with associated haemorrhage. Areas of lymphocytic infiltration are present (Fig. 7 ; photomicrograph Rat 77).

*Reticulum cell sarcoma.*—One rat died five months after intratesticular implantation, apparently from intestinal obstruction, and two rather haemorrhagic tumour nodules of reticulum cell sarcoma were present in the mesentery of the ileum. Its right testis, fixed to the lower part of the psoas muscle by adhesions, showed degenerative changes and fibrous encapsulation of the pellet.

*Control experiments—methyl-cholanthrene series*

The carcinogenic activity of these methyl-cholanthrene-cholesterol pellets has been borne out by the high incidence of tumours produced subcutaneously in eight out of twelve rats inoculated. Table III shows that of these eight sarcomata, all developed at least one year after inoculation of the pellet. Histologically these tumours were spindle cell growths with pleomorphic areas and moderate numbers of mitoses. No metastases were found grossly or microscopically at the time of death, although locally there was invasion of muscle and skin. Fifteen rats in which the methyl-cholanthrene pellets had been present in the testes for 18 to 21 months had the pellets extracted, washed and implanted subcutaneously in new hosts of the same strain 4 months old. The rats, approximately equal numbers of either sex, developed two subcutaneous fibro-sarcomata at 5 months, after which all had to be killed. One of these developed in each sex. It is interesting that all these pellets at the last necropsies, 26 months after original implantation, still gave a strong yellowish-green fluorescence under ultra-violet light, thus indicating that an appreciable quantity of methyl-cholanthrene remained unabsorbed, and that in the testis, even the connective tissue is slow to be affected by this substance.

TABLE III.—20-*Methyl-cholanthrene Control Series. Implantation into Subcutaneous Tissues of Loin of Pellets as used in Previous Experiments.*

| Series X. | Numbers involved. | Number developing tumours at site of pellet. | Age at date of inoculation. | Age when tumour noted— 12–18 months. | 18–24 months. | 24–30 months. |
|---|---|---|---|---|---|---|
| Male | 6 | 3 | 6 months | — | — | — |
| Female | 6 | 5 | 6 ,, | — | — | — |
| Total | 12 | 8 | — | 1 | 3 | 4 |

*2-Acetyl-amino-fluorene series*

Sixteen male rats, aged 4 months, had intra-testicular inoculation of this carcinogen as described above. One, Rat 137, died after 10 months, the others were killed after 14 months.

In 2 of the 16 animals injected with 5 mg. of the carcinogen in 0·2 ml. of tricaprylin, tumours of the interstitial cells of Leydig arose in the inoculated testis. In one, Rat 137 (Fig. 8), there was in addition to the testicular tumour diffuse infiltration of the peritoneal cavity by tumour nodules with invasion of the kidneys and liver. The testicular tumour, like the other interstitial cell tumours, contained lipoid-laden cells with few mitoses, but in places the cells were more spindle-shaped. The peritoneal and hepatic tumours were seen to be rather pleomorphic sarcomata with numerous mitoses. Only a few of the tumour cells contained lipoid. The reticular pattern of the testicular and peritoneal growths is somewhat different (Fig. 9*a*, *b*—photomicrograph Rat 137). In the other case of interstitial cell tumour, Rat 138, there were no other features. In both these cases and in the other 14 rats which eventually came to necropsy, there was fibrous encapsulation of the inoculum, which had darkened considerably. There was some interstitial cell hyperplasia and patchy degeneration and calcification of tubules.

*Zinc salts*

Results are tabulated in Table IV.

TABLE IV.—*Aqueous Zinc Salt Injections Into Right Testis of Rats*

| | Inoculum. 0·15–0·20 ml. of 10% zinc sulphate. | 0·15 ml. of 5% zinc chloride. |
|---|---|---|
| Number of rats | 19 | 29 |
| Age of inoculation (months) | 4 | 3 |
| Age when killed (months) | 19 | 18 |
| Testicular tumours | 0 | 0 |
| Changes in inoculated testis | All showed extensive hyalinisation of tubules with several tubules calcified. In a few seminiferous tubules Sertoli cells remained with degenerate spermatozoa. Interstitial cells present in moderate numbers. | |

One rat—Rat 150—died 8 months after inoculation and was found to have a large retro-peritoneal fibro-sarcoma arising near the pancreas. Another died of leukaemia 18 months after inoculation. This rat showed enlargement of spleen and mesenteric lymph nodes, infiltration of liver and primitive white cells in the peripheral blood.

*2-Amino-fluorene*

The 9 rats injected with this carcinogen locally into the testis showed no malignant testicular changes after 6 months when all were killed. The inoculum was encased in fibrous tissue and many tubules showed degeneration and calcification.

### DISCUSSION

Experimental Series III of 15 rats (Table II) proved the most productive. Young male rats 1 month old were employed, the testis was fixed intra-abdominally at that age, and soon after, before notable testicular changes could occur, the implantation of carcinogens was carried out. Seven of these animals survived to 2 years of age and the two malignant tumours which developed arose at an age of 1 year 10 months and 1 year 6 months. Neither of these tumours bore any resemblance to human seminomas, being spindle cell sarcomas closely similar to those produced in the subcutaneous sites (Fig. 5 and 7). They do not correspond to Sertoli cell tumours, which are usually alveolar in structure and they bear no resemblance to the tumours of the interstitial cells of Leydig, which appeared in other rats. The only proliferation of fibroblasts seen in the sections of inoculated testis was in the immediate capsule of the pellet and it would seem more likely that these too neoplasms arose from these, from the slightly thickened testicular capsule, the scanty connective tissue stroma, or the adjoining abdominal wall. The last mentioned origin was unlikely as invasion of the abdominal musculature was early and the growths were mainly intra-abdominal in site. The methyl-cholanthrene would appear to have been very slowly absorbed from the pellet and subsequent calcification of its shell might have impaired further absorption, but the continuing potency is well borne out by the carcinogenic activity in a new subcutaneous site as shown in Table V. This would seem to indicate that the small amount of interstitial connective tissue in the testes responds very slowly and only rarely to this potent carcinogen, compared with the subcutaneous connective tissue which responds frequently and in a much shorter time (Tables IV and V). Both the tumours arising at the site of the intra-abdominal testis appear to have destroyed it almost completely, only a few tubuli recti remaining in one. The question of their origin from the interstitial cells must be considered. It is accepted by many that the interstitial cells of Leydig are modified fibroblasts. The characters peculiar to these cells appear in their hyperplasias and neoplasms and also in their metastases, although Masson and Sencert (1923), reporting a malignant interstitial cell tumour in a man aged 62 years noted that the metastases occurring in the nodes, abdominal wall and lungs showed a more uniform cellular structure, quite different from normal Leydig cells. Their diagrams show, however, a clear cell structure. Innes (1942) describes interstitial cell tumours in animals and comments on their vascular nature ; the two intra-abdominal testicular tumours showed none of these features.

The significance of the tubular changes in Rat 20 are difficult to assess. In an epithelium so primitive and undifferentiated as the seminiferous tubules and so liable to degeneration it is not easy to decide when pre-malignant change has occurred and although these appearances (illustrated in Fig. 4) are probably a sequal to intra-abdominal fixation of the testis or to methyl-cholanthrene, it is by no means certain that they would have progressed to frank intra-tubular carcinoma. It was thought that the combination of experimental cryptorchidism closely mimicking the natural defect in humans and the implantation of carcinogenic pellets might have produced a small number of testicular tumours. As the pellet had so little destructive effect on the testis, it was not considered worth while to see the effect on a large scale of intra-abdominal fixation alone. The production of testicular sarcomas in 2 rats and atypical tubular changes in another occurred in the 40 rats with an abdominal testis, while the results in the 79 rats with normally sited testes implanted with methyl-cholanthrene were completely negative. The numbers, however, are too small to be of statistical significance. They do not show any noted tendency of the testis when artificially placed within the abdomen at an early age to undergo malignant change. In the human cryptorchid, hormonal control of the descent is generally believed to be abnormal, and in that respect the experiments performed to date do not reproduce exactly the same set of circumstances. As previously mentioned the processes vaginalis remains patent in the rat and natural cryptorchidism with a non-functioning testis would appear to be extremely rare.

The 6 cases of interstitial cell growth show histological appearances identical with previous cases reported in the literature. That none occurred in the 48 rats of a different colony injected with zinc salts may be of some significance, but the number of cases is too small to draw any conclusion. The tumours or hyperplasias of interstitial cells were discovered at autopsy, and were therefore found mainly in the older animals. In Rats 137 and 138, injected intra-testicularly with 2-acetyl-amino-fluorene at 5 months and dying at 1 year 11 months, the Leydig cell growths appeared in the inoculated testes—the only instance in which this occurred. Statistical analysis shows a reasonable probability that they have arisen by chance in the inoculated rather than the untreated testis. The probability level is 0·50. The omental and hepatic growths described in Rat 137 are largely spindle cell growths with pleomorphism in places, although a few lipoid laden cells are present. It was considered, however, despite Masson and Sencert's case (1923) that the appearances did not justify identifying these growths with the benign-looking adenoma in the testis, although such a possibility could not be excluded (Fig. 9).

### SUMMARY

The experimental attempts in the past 30 years to induce in animals testicular neoplasms comparable to those arising spontaneously in man have been reviewed.

A fresh attempt has been made to induce tumours in the rodent testis in the hope of providing material for studying the histogenesis of the common human types, especially the seminoma. 20-Methyl-cholanthrene-cholesterol pellets, 2-acetyl-amino-fluorene, 2-amino-fluorene, zinc chloride and zinc sulphate solutions have been employed at different age periods and with methyl-cholanthrene, a proportion of the animals had intra-abdominal fixation of the injected testis.

In the experiments which have been described, two fibro-sarcomatous tumours, one with metastases, arose at the site of the intra-abdominal testis. The possible origin of these from different testicular or extra-testicular tissue is discussed. These tumours arose in a group of 15 rats in which the testis was fixed intra-abdominally at one month of age and implanted with a methyl-cholanthrene-cholesterol pellet at one month later. In the same group one animal showed possible pre-malignant change in the seminiferous epithelium. In the 119 rats of the methyl-cholanthrene series, 4 interstitial cell growths appeared in the opposite testis : in 2 of these the inoculated testis had been intra-abdominal. The relationship between functional activity and malignancy in the naturally occurring disease and in experimental work is discussed.

No interstitial cell growths arose in 48 rats from a different colony inoculated with zinc salts.

Two interstitial cell growths arose in the inoculated testis of 2 rats 18 months after inoculation of 2-acetyl-amino-fluorene. These rats, members of a group of 16, were 2 years old at time of post-mortem examination. One had multiple fibro-sarcomatous tumours with clear cell areas throughout its peritoneal cavity and in the liver. These numbers are too small to attach significance to origin in the inoculated side.

The results obtained by inoculation of zinc salts and 2-amino-fluorene were essentially negative in producing testicular malignancy.

I should like to acknowledge my indebtedness to Professor D. F. Cappell for his advice at all stages of this experimental work and his criticism of the manuscript ; to Professor T. Crawford for all the photo-micrographs.

My thanks are due to the technical staff of the Department of Pathology at the University and Western Infirmary, Glasgow and St. George's Hospital Medical School, London, for many of the histological preparations ; to Mr. Kerr of the Department of Pathology, Glasgow University, and also the Photographic Department of St. George's Hospital, London, for the photographs of gross specimens.

Bagg, H. J.—(1936) *Amer. J. Cancer*, **26**, 69.
Bell, F. G.—(1926) *Brit. J. Surg.*, **13**, 282.
Carrol, W. A.—(1949) *J. Urol.*, **61**, 398.
Champy, C. and Laveden, J. P.—(1939) *Bull. Ass. franç. Cancer*, **28**, 503.
Cook, J. W.—(1935) *J. Amer. chem. Soc.*, **57**, 1380.
Curtis, M. R., Bullock, F. D. and Dunning, W. F.—(1931) *Amer. J. Cancer*, **15**, 67.
Dixon, F. J. and Moore, R. A.—(1953) *Cancer*, **6**, 424.
Gardner, W. U.—(1943) *Cancer Res.*, **3**, 757.
Gillman, J., Gilbert, C., and Spence, I.—(1953) *Cancer*, **6**, 464.
Gullota, G.—(1953) *Bull. Soc. Méd. Chir. Catania* **7**, 129.
Hartmann, Menetrier, Peyron, and Isch-Wall.—(1922) *Bull. Ass. franç. Cancer*, **11**, 319.
Ilfeld, F. W.—(1936) *Amer. J. Cancer* **26**, 743.
Innes, J. R. M.—(1942) *J. Path. Bact.* **54**, 485.
Isaka, H.—(1951) *Gann.* **42**, 351.

KENNAWAY, E. L.—(1930) *Biochem. J.* **24**, 497.
LACASSAGNE, A.—(1933) *C.R. Soc. Biol. Paris*, **114**, 660.
MAISIN, J.—(1948) 'Cancer: Hérédité, hormones, substances cancérigénes', Paris. (Castermann)
MASSON, P. AND SENCERT, L.—(1923) *Bull. Ass. franç. Cancer*, **12**, 555.
MICHALOWSKY, I.—(1928) *Virchows Arch.*, **267**, 27.—(1930) *Ibid.*, **274**, 319.
NICOD, J. L. AND REGAMEY, J.—(1938) *Bull. Ass. franç. Cancer*, **27**, 706.
SPINELLI, A.—(1941) *Tumori*, **27**, 367.
SPAMPINATO, V.—(1951) *Arch. ital. Anat. Istol. patol.*, **24**, 357.
WILLIS, R. A.—(1934) *Brit. J. exp. Path.*, **15**, 234.